# Building resilience to food insecurity in rural communities: Evidence from traditional institutions in Zimbabwe

**DOI:** 10.4102/jamba.v9i1.453

**Published:** 2017-06-30

**Authors:** Emmanuel Mavhura

**Affiliations:** 1 Department of Geography, Bindura University of Science Education, Zimbabwe

## Abstract

Many rural communities that depend on smallholder farming face food insecurity induced by climate-related disasters. In response, some communities are taking the initiative to cope and adapt to climate-related disasters. Using case study material from the Zambezi Valley, Zimbabwe, this article examines how traditional institutions are enhancing resilience to food insecurity in rural areas. The data were collected through interviews and focus groups involving traditional leaders, ward councillors, village civil protection members and villagers selected in the valley. The findings point to how the Zunde raMambo informal safety net, nhimbe form of collective work and the practice of share-rearing arrangement to access draught power help save lives and alleviate food insecurity induced by flood or drought disasters. The study concludes that the three schemes are evidence of community reorganisation or change in response to food insecurity. They are a form of absorptive capacities enabling the community to cope with food insecurity.

## Introduction

Resilience is an increasingly common concept throughout a range of research domains, particularly in relation to shocks, economic downturn, climate change, globalisation and environmental disasters (Skerratt 2013; Wilson 2013). Engineering science conceptualises resilience as the resistance of buildings and critical infrastructure. This approach considers resilience as an outcome measure with an end goal of limiting damage to infrastructure, mitigating the consequences, and recovery to the pre-disaster state (Cutter, Burton & Emrich 2010). Thus, resilience in the engineering community is associated with robustness and ductility of building materials (Fekete, Hufschmidt & Kruse 2014). It is also viewed as a form of buffering in order to maintain a basic structure. However, understanding resilience as buffering may prevent necessary changes that would enable more sustainable development. This is particularly true when considering the role of institutions in building community resilience. At the same time, a return to pre-disaster conditions can propagate the conditions of vulnerability that can lead to the disaster (Jordan, Javernick-Will & Amadei 2011). In view of these shortcomings, this article adopts the social-ecological perspectives in which resilience is understood as the ability of a community to recover from disasters through adaptive processes that facilitate the social system to reorganise, change and learn in response to a disaster (Coetzee, Van Niekerk & Raju 2016). This is supported by Cutter et al. (2008) who view resilience as the ability of a social system to respond and recover from disasters and includes those inherent conditions that allow the system to absorb impacts and cope with the event. In this way, resilience is conceptualised as a process of transformation and not as ‘buffering’ as this will lead to the reinforcement of existing practices and prevent the questioning of underlying assumptions and power relations.

Conceptually, resilience is closely related to vulnerability. Some scholars view resilience as ‘ability to’ and vulnerability as ‘inability to’, thus making the two terms opposite of each other (Birkmann 2006; Cutter et al. 2010). On the one hand, resilience building can increase capacities to prepare for impending hazards (Lei et al. 2014), thereby reducing vulnerability to disasters. On the other hand, vulnerability reduction contributes to resilience building through reducing exposure, reducing sensitivity and strengthening institutional capacities (Lei et al. 2014). This is especially important in resources-constrained rural places.

Disaster risk reduction initiatives encapsulate the growing recognition that building community resilience is the key to reducing the impact and severity of disasters (Fois & Forino 2014). Efforts to build community resilience can be complicated but should be community-driven, with clear goals and priorities for what an individual community considers necessary to become more resilient. One of the inherent complexities of building community resilience is that goals can differ among communities because communities must tailor the outcomes to their individual needs. In Pakistan, Ainuddin and Routray (2012) found that communities were the first responders to earthquake disasters that occurred between 1954 and 2004. Coates (2015) observed that communities are dynamic and respond to changes that may occur in population, politics, economy and the environment. Those communities with a strong economy, commitment to social justice and strong environmental standards may be able to ‘bounce back better’ and move forward after a disaster. Building resilience of such communities may depend on the ability of an affected community to monitor change and then modify its plans and activities appropriately to accommodate the observed changes.

In Zimbabwe, recent studies have indicated that climate variability and change are already having an adverse impact on rural communities who mainly depend on smallholder farming (Mavhura, Manatsa & Mushore 2015). Drought, changing seasons, erratic rainfall patterns, heavy rainfall, and strong winds are among the main climate-related disturbances experienced by local people. Although some individuals have developed coping strategies, there is a limit to the extent of broader community resilience that can be fostered through such individual efforts (Brown & Sonwa 2015). Studies conducted in Zimbabwe showed that by 2016, the *Civil Protection Act* and the *principal disaster act* in Zimbabwe had no room for community participation in the prevention and mitigation of disasters (Mavhura 2016). Given the importance of traditional institutions in influencing the adaptation of rural households, this study was conducted at the community level in the Zambezi Valley, Zimbabwe, to examine if and how traditional institutions were enhancing resilience to food insecurity in rural areas.

This study contributes to an emerging literature in disaster studies on the importance of traditional institutions and practices as enhancing disaster risk reduction (Berman, Quinn & Paavola 2012; Brown & Sonwa 2015; Rumbach & Foley 2014). Despite a growing interest in traditional knowledge in fields such as agriculture and medicine, mainstream disaster science has marginalised traditional practices (Rumbach & Foley 2014). This case study points to how informal safety nets, collective work and the practice of share-rearing of draught power help save lives and improve food security induced by climate-related disasters.

After this introduction, this article discusses the role of traditional institutions in building disaster resilience. It then describes the study area and methods used to gather data. Later findings are presented, followed by their discussion before the conclusion in the last section.

### Institutions and disaster resilience

The term institution is used in relation to organisations, human relationships and/or rules governing the behaviour of people (Banerjee et al. 2012). The World Bank (2000) defines the institution as a set of formal and informal rules governing individuals and organisations. Formal institutions include organisations with a legally defined role, structure, and in some cases, sets of procedures. Informal institutions include social networks, associations, conventions and codes of behaviour (Kayaga, Mugabi & Kingdom 2013). The informal institutions may also have structures and sets of procedures, but these may have no legal or written basis.

While institutions can enable and maintain certain practices, they can exclude certain actors or constrain the same practices (Berman et al. 2012). As a result, the way in which individuals behave and interact with each other, combined with the policies and processes that are determined by external agents, will influence how any one individual is able to respond to a particular hazardous event. Some institutions regulate the dynamics of market systems, local governance of common-pool resources, land tenure and access, all of which are important elements for rural disaster resilience (Berman et al. 2012). Other institutions are often interlinked and shape not only how households and communities are impacted by hazards, but also how they respond to the disasters (Brown & Sonwa 2015). During the pre-disaster period, institutions may build livelihood assets, improve household production and incomes, and enhance risk coping strategies. In the relief phase of disasters, some institutions focus on search and rescue as well as meeting basic needs such as shelter, water and food. Later in the rehabilitation stage, the goals of institutions may include preventing further erosion of productive assets, strengthening coping strategies and helping households re-establish their livelihoods. In certain circumstances, institutions may link local systems to larger spatial systems that enhance resilience (Berman et al. 2012).

Concerns have been raised about the paucity of efficient and flexible institutions in disaster risk reduction (Ali & Jones 2013; Gopalakrishnan & Okada 2007). Weak institutions have been blamed for the lack of disaster resilience in many African countries. In the Congo Basin forests of Cameroon, Brown et al. (2010) observed that weak linkages among government institutions reduced the level of adaptive capacity to climate change. Weak institutions have also reduced the level of development and consequently resilience by creating gender differences in African societies (Kumssa & Mbeche 2004). For example, the family institutions in African societies determine the division of labour among individuals, where in some communities the wife and the children are the only people who toil in the fields. Some cultural values and traditions also deny educational opportunities to the girl child.

Despite the burgeoning literature, however, very few studies have focused on how traditional institutions can build resilience to food insecurity induced by disasters such as drought and flood. Traditional institutions are generally held in high esteem in many rural communities of Africa including Uganda, South Africa and Zimbabwe (Ellis & Bahiigwa 2003; Manyena 2014; Tandlich, Chirenda & Srinivas 2013). Manyena (2014) posits that traditional institutions are formal and informal structures of (re)building social networks that provide a sense of normality and stability before, during and after a disaster. In Zimbabwe, traditional institutions are made up of traditional leaders including the chiefs, headmen and village heads.

The Zimbabwean government enacted the *Traditional Leaders Act* (Chapter 29:17) to regulate the activities of traditional institutions (Government of Zimbabwe 2001). The duties of traditional leaders include notifying the local authority for the area concerned of any natural or human-induced disasters affecting the inhabitants, livestock, crops, land, flora and fauna (Government of Zimbabwe 2001). Traditional leaders are also involved in the coordination of disaster relief operations in their areas. To achieve these tasks, chiefs are assisted by headmen and village heads. While headmen preside over ward assemblies when elected as chairperson for those wards, village heads preside over the village assembly (*dare* in Shona or *inkundla* in Ndebele) in ensuring that all lawful and reasonable orders of the chief or headman are adhered to. However, the delegation of any functions by the chief does not divest the chief of that function, as he may revoke at any time any order given by a headman or village head in the exercise of that function (Manyena 2014).

The capacity of traditional institutions to build disaster resilience depends to a large extent on its social capital. Social capital includes social networks, connections, membership of groups, relationship of trust, norms and any other social resources upon which people draw in pursuit of livelihood objectives (Nyamwanza 2012). Notwithstanding that traditional institutions may be a driving force of social cohesion and contribute to creating and protecting social capital and livelihood, they may also place constraints upon specific groups of people such as women, children and the disabled (Manyena 2014). This may render traditional institutions incompatible with the goals of formal state institutions. For example, a study conducted in the Chipinge district (Zimbabwe) showed that chiefs were discouraging the use of condoms that the government was promoting to combat the HIV and AIDS pandemic, although they encouraged people to abstain from premarital and out-of-marriage sex (Marashe 2014).

Social capital can positively and negatively affect community resilience to disasters (Coates 2015). On the one hand, interpersonal trust and networks may provide important resources for coping with a variety of disasters. On the other hand, closely-knit communities may exclude, reject or deny potential people from affiliating and benefiting from community programmes that enhance resilience to disasters. Allowing a few people to enjoy the benefits of closely-knit communities jeopardises the resilience of the excluded, denied or rejected group(s). This is because the denial and or rejection of other people may damage the stability of that particular group. For example, the majority of some communities may be members of a single clan. In such situations, arable land might be open to any villager, but the distribution of farming inputs might be limited to the members of that particular clan. Other households that do not belong to the clan may be considered aliens and excluded from benefiting from drought relief programmes despite being vulnerable. Their social networks within the community may be weak as well. This would weaken their resilience to drought. Manyena (2014) observed that the potentially negative consequences of closed social networks widen the distinction between bonding and bridging effects of social capital. The bonding effect of social capital occurs when social networks result in the distribution of benefits within communities but remaining closed to outsiders. The bridging effect of social capital happens when networks contribute to cross-cultural and intergroup linkages. Such linkages have the potential to generate far more positive outcomes that benefit different communities exposed to disasters. Resilient communities that lack bridging social capital may, therefore, create greater cohesion and enhanced resilience at a micro level. However, this may contribute to more dangerous forms of exclusionary and competitive politics at a macro level that result in increased vulnerability to hazards (Manyena 2014). The effect of social capital on community resilience has remained understudied.

### Study area

**FIGURE 1:**
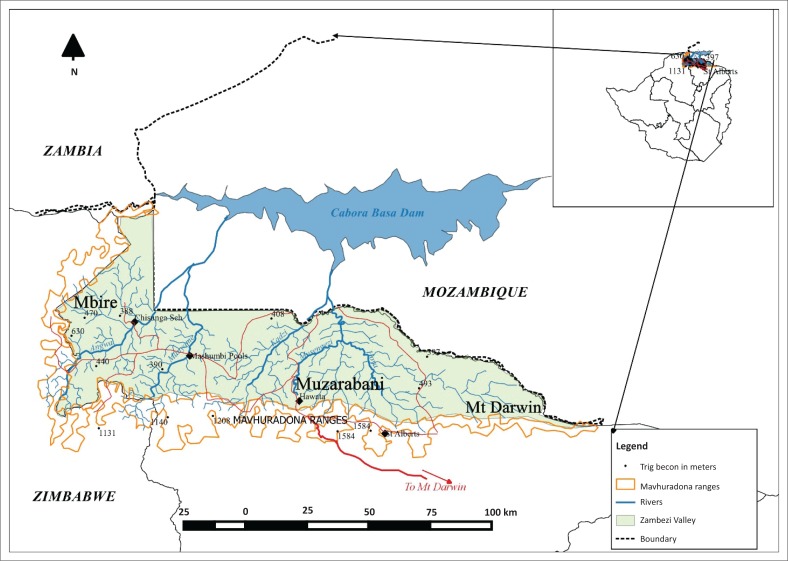
Map of the Zambezi Valley, Zimbabwe.

This article uses case study materials from the Zambezi Valley in northern Zimbabwe. The specific area of the valley includes three districts: Mbire, Muzarabani and Mt Darwin (Figure 1). The Zambezi Valley is a flat terrain of about 300 m above sea level. This terrain makes it susceptible to tropical cyclones originating from the Indian Ocean. Rivers that originate from the high veld, dissect the valley and flash flood coming from the Mavhuradonha Mountain Range are common (Madamombe 2004). The soils over much of the valley are sodic, and specialised vegetation communities have adapted to the highly mineralised soils. The vegetation that predominates is *Colophospermum mopane* (known as mopane woodlands). There are also pockets of ecologically important dry forests including *Acacia* spp., *Commiphera* spp. and baobab.

The Zambezi Valley is a typical rural area in Zimbabwe that is very remote and under-developed. The area was chosen for two main reasons. Firstly, the valley is among the most flood-prone areas in Zimbabwe. Two types of flood have been affecting the valley for decades. The first and most frequent is the seasonal flood, which frequently occurs in January or February, at the peak of the rainfall season (Mudavanhu et al. 2015). This usually results in backflow along major rivers including Musengezi, Hoya, Nzoumvunda, Angwa, Dande and Hunyani. The second and not so frequent one is the cyclone-induced flood. In 2000, Cyclone Elin induced floods in the Zambezi Valley and other eastern districts of Zimbabwe that left 120 people dead, over 250 000 people affected, and approximately $7.5 million in economic losses (Mavhura et al. 2013). The flood caused great damage to houses, crops, electricity supply lines and food stocks. They also promoted the spread of diseases such as malaria and cholera. Economic activities were disrupted, thereby creating financial stress on the poor people.

Secondly, the Zambezi Valley is characterised by food insecurity–induced drought. It is located in agro-ecological region IV that experiences low annual precipitation of 450 mm – 650 mm, seasonal droughts and severe intra-season dry spells (Mavhura et al. 2015). The rain season is unimodal, extending from mid-November to the end of March. Precipitation typically occurs on a number of isolated days and locations, seldom exceeding 50 rain days per annum. Mudavanhu et al. (2015) posit that the climate of the valley is largely controlled by global atmospheric circulation patterns, the most important among them being the movement of the inter-tropical convergence zone that determines the annual seasonality of precipitation across tropical Africa. Mavhura et al. (2015) found high variability of rainfall in the valley that makes the area more susceptible to drought. The onset and cessation dates of rainfall are barely reliable, as they can vary by as much as 23 and 27 days, respectively.

According to Madamombe (2004), small-scale rain-fed agriculture is the main source of livelihood in the Zambezi Valley. Crops grown are maize, small grains, cotton and tobacco. The yield levels are very low especially during years of severe drought. In some cases, a little surplus is realised which is then saved for other household needs. Livestock rearing is also practised at subsistence level (Madamombe 2004). In response to the low yields, smallholder farmers diversify their sources of income by engaging in petty business. The diversification is also a way to accumulate wealth. However, the security of the livelihoods of the smallholder farmers remains closely linked with the productivity levels of the local agro-ecological zones, which are hindered to a large extent by water availability (MEA 2005; Stringer et al. 2009).

## Methods

Data for this study mainly came from 50 interviews held in 2016 with key informants involving traditional leaders (3 chiefs, 5 headmen, 8 village heads), 10 ward councillors, 6 disaster practitioners, 15 villagers and 3 academics engaged in disaster risk reduction in Zimbabwe. The key informants were randomly selected across the Zambezi Valley depending on their availability and willingness to participate in the study. Open-ended questions were used to allow the participants to respond in their own words rather than forcing them to choose from fixed responses as closed questionnaires do (Mack et al. 2005). The open-ended questions evoked responses that were meaningful and culturally salient to the participant and rich and explanatory in nature. The duration of each interview ranged from 1 to 2 h, depending on the respondent. The interviews were mainly semi-structured to allow the language of the interview to be adapted to the ability and educational level of the respondents (Majumdar 2011).

The data also came from six focus group sessions held in 2016. The focus groups were made up of community members purposively selected from villages across the three districts in the valley. Two focus groups were conducted in each district. The selection was based on their active role in community development and included traditional leaders, ward councillors, village civil protection members and people living with HIV and AIDS. The focus groups produced more filtered, ‘socially controlled’ and more neutral findings (Cohen et al. 2011).

The raw data from interviews and focus group discussions underwent thematic analysis (Patton 2002). This is a recognised analytical approach in qualitative research. Three key themes emerged: (1) informal safety nets, (2) collective work and (3) draught power. All responses for each theme were then examined to identify any areas of consensus and differences (Skerratt 2013). Patterns, connections and relationships among the themes were identified. The emphasis of this approach was on richness, diversity and complexity rather than solely trying to identify consensus within themes (Denscombe 2010; Skerratt 2013). The findings were reported under the identified themes to show how the traditional institutions enhanced resilience to food insecurity through their abundant social capital.

## Findings

This section focuses on how informal safety nets, collective work and share-rearing arrangement of draught power enhance resilience to food insecurity in rural communities of Zimbabwe.

### How informal safety nets enhance food security?

Social capital is a critical arm of traditional institutions in the Zambezi Valley, Zimbabwe. It is abundant and innovative, including informal safety nets, social networks, connections, relationship of trust and other social resources upon which people draw in pursuit of livelihood objectives. Informal safety nets play a crucial role in villages where official government support is very limited or non-existent. One of the informal safety nets used by traditional institutions is the *Zunde raMambo* scheme that aims at developing self-sustenance of the community and reducing the vulnerability of people through the provision of food. The term *Zunde raMambo* is a local phrase that literally means ‘the chief’s granary’. It is a traditional social security arrangement designed to protect vulnerable groups: widows, orphans, the sick, the elderly and those affected by disasters such as flood and drought. This arrangement provides a platform for collective action by community members. Chiefs allocated land for collective production of cereals for needy households. This common land is the *Zunde*. Members of the community provide labour on a voluntary basis taking turns to participate in the production process including ploughing, sowing, weeding and harvesting. One village head reported:

The harvest is stored in granaries at the chief’s homestead which is then distributed to the chief’s subjects only in the event of food shortages. (Male village head)

The *Zunde raMambo* scheme has been decentralised to villages, where each village head has allocated land for cultivation by the community. The scheme has a leadership structure incorporating the village head as chairperson, a secretary and a treasurer, all chosen by the community. For the community:

Volunteering means contributing farming inputs towards the scheme, and giving up one’s time to work in the fields for the benefit of the less privileged members of the community. (Male village head)

Table 1 shows the statistics of land under the *Zunde raMambo* scheme in one of the sampled wards, Dambakurima Ward of Muzarabani, and the harvest realised between 2011 and 2014. The villagers used a roster to participate during weeding, harvesting and post-harvest processing of food crops. The initiative provides a platform for interaction among smallholder farmers and traditional leaders who help to mobilise communities, create confidence through the *Zunde raMambo* concept and enhance resilience to food insecurity induced by drought and flood disasters. Participants of the scheme realise the importance of building local-level food reserves to cushion the vulnerable groups during drought or flood disasters.

**TABLE 1:** Crop production of the *Zunde raMambo* scheme (2011–2014).

Year	Ha of land under cultivation	Crop	Production (tonnes)
2011	10	Maize	11.0
	5	Sorghum	3.0
2012	9	Maize	10.0
	6	Sorghum	2.5
2013	9	Maize	10.0
	8	Sorghum	4.0
2014	9	Maize	10.0
	8	Sorghum	3.5

Ha, hectarage.

Crops grown in the scheme are mainly cereals in the form of maize and sorghum that form the staple food. Cash crops such as cotton and tobacco are not grown under the *Zunde raMambo* scheme, although such crops are also grown by individual households. This may indicate that the purpose of the *Zunde raMambo* scheme is to provide food to the needy and not raise cash. Two days are set aside per week for working in the *Zunde raMambo* scheme in each village.

There are two main advantages associated with the *Zunde raMambo* scheme. The majority of the participants (88%) felt that community cohesion increases when people work together in a *Zunde raMambo* scheme. This is very important for the quick recovery and individual’s capacity to withstand stressors and not manifest psychology dysfunction, such as mental illness or persistent negative mood, in the face of flood shocks and disturbances. The other advantage is the availability of large reserves of food that enable affected households to return to their status quo after flood or drought events. In 2012, floods struck all low-lying areas in flood plains of the valley and washed away crops of about 10 000 households. The affected households faced critical food shortages until they got assistance from the *Zunde raMambo* proceeds. However, this does not necessarily mean that the *Zunde raMambo* scheme does not face challenges. For the scheme to be effective, there are several constraints requiring attention. For instances, it is reported that in 2012, two tonnes of maize were destroyed by pests in one ward because of lack of grain protectants. Moisture also destroyed other grain during the 2014–2015 rainy season because of inadequate storage facilities. This then resulted in food insecurity in the community. Furthermore, the prohibitive costs of inputs compromise the effectiveness of the *Zunde raMambo* scheme. This has resulted in low application of inorganic fertilisers, limited use of high yielding varieties, herbicides and insecticides leading to low output. Flood also reduces the farming output of *Zunde raMambo by washing away crops along flood plains*. They also trigger the spread of malaria, diarrhoea and cholera that reduce the productivity of the villagers taking part in the *Zunde raMambo* scheme. Shortage of draught power forces community members to give first priority to till their land. This results in late planting of crops under the *Zunde raMambo* scheme, which ultimately reduces output.

Despite these challenges, the Zambezi Valley community holds the *Zunde raMambo* scheme in high esteem. The scheme is found in each ward assembly studied. The majority of the key informants (90%) agreed the *Zunde raMambo* scheme is still necessary as a local initiative to cushion food insecure households. They explained that the *Zunde raMambo* scheme was more sustainable than food-for-work programmes from government and food handouts from non-governmental organisations (NGOs). Many people (95% of the participants) are participating in the *Zunde raMambo* scheme. The traditional leaders mobilise the villagers volunteer their labour and inputs for the welfare of the vulnerable groups including orphans, food insecure households, the sick and the elderly.

Apart from the *Zunde raMambo* scheme, smallholder farmers in the Zambezi Valley have networks with both bonding and bridging effects on the communities. The bonding effect of social capital is evidenced by the distribution of grain under the *Zunde raMambo* scheme among disaster victims and other vulnerable members across communities. The bridging effect happens when their networks cut across tribal, religious and political divides when faced with food insecurity. About 70% of the focus group participants admitted that households whose crops were not destroyed by flood in 2012, (partly because they were outside the flood plains), assisted their counterparts with maize and sorghum that constitute the staple food in the valley. Others either sold their grain at affordable prices to flood victims or, lent them the grain they needed until the next season. Therefore, social capital is strengthening community resilience both at micro and macro level.

### Role of collective work in building resilience to food insecurity

Apart from the *Zunde raMambo* scheme, rural communities in Zimbabwe employ collective work locally known as *nhimbe*, to assist households without draught power and farming equipment. The *nhimbe* is organised at village level where one household invites others to provide labour and draught power for use during activities such as ploughing, planting, harvesting and threshing. The host prepares food, refreshments and beer for consumption while the invitees work on the task called for. This ensures that farming processes are done on time. The invited guests are not paid for the work. On the one hand, *nhimbe* strengthens social cohesion by bonding and bridging households within and across the Zambezi Valley. In 2011 for example, village heads in Dambakurima Ward called for five groups of *nhimbe* that lasted for 10 days each of land preparation and planting of seeds. This resulted in the preparation and planting of 2 ha of land under maize production for almost every household in the concerned villages. Planting in time enabled the maize crops to mature within the short rain season. This ultimately reduces vulnerability and enhances resilience to food insecurity induced by drought disasters.

On the other hand, when adequate rainfall, seeds and fertilisers are available, the *nhimbe* arrangement enhances food security among the poor households by providing farming equipment, draught power and timely farming processes including planting, weeding and harvesting. The *nhimbe* scheme also acts as a platform for networking with members sharing expertise, skills and experiences in dealing with food insecurity. Key informants cited the construction of grain storage facilities that are raised from the flood-predicted levels during the *nhimbe* schemes. Others reported some conservation farming techniques and rainwater harvesting as activities that are shared during the *nhimbe* schemes. Both conservation farming and rainwater harvesting enable communities to improve food security in the Zambezi Valley.

### Community arrangement to access draught power

Drought power in rural areas that depend on smallholder farming is very important in reducing vulnerability to and enhancing resilience to drought disasters. In Zimbabwe, some smallholder farmers without draught power use some institutional arrangements to access them. Firstly, there is a ‘share-rearing’ arrangement in which the livestock are fully owned by one person but raised by a different household. For example, a family without draught power may request to take care of livestock belonging to families with large heads of cattle. The carer is not paid for this task. Instead, the carer uses the cattle for draught power whiles keeping the manure and drinking milk. This arrangement is usually found between close relatives and friends. The ‘share-rearing’ arrangement depends on the strength of social capital possessed by a household within the community. People with many social networks can easily make ‘share-rearing’ arrangements and enjoy the related benefits. This is enabling households without farming implements to prepare their land for crop production.

The second arrangement is a short-term one which involves using cattle for a particular task: ploughing. The poor households access draught power on an exchange basis. One or 2 days of using the cattle are exchanged for 1 or 2 days for human labour. This arrangement enables the poor households to use livestock they do not own. The arrangements for accessing draught power show the existence of a high degree of social capital with bonding effect among the smallholder farmers in the Zambezi Valley.

## Discussion

Traditional institutions mediate the human–environment relations when faced with a hazard (Manyena 2014). Resilient traditional institutions tend to be characterised by the capacity of people to deal with complexity, uncertainty and interplay between slow-on-set and rapid-on-set hazards. The Zimbabwean chieftaincy is one of the traditional institutions that has authority and capacity to mobilise people for collective action (Manyena 2014). The chiefs are assisted by headmen and village heads in overseeing their areas of jurisdiction. Manyena (2014) posits that traditional institutions have adapted to changes through social learning which is stored in the memory of individuals and communities particularly the elderly. As Brown and Sonwa (2015) argued, traditional institutions can create flexibility in problem-solving, thereby increasing people’s adaptability and resilience to disasters. However, this type of institutional memory may be subject to erosion when the leaders who are usually the elderly, fall ill or die, making them non-sustainable.

The analysis of the traditional institutions in enhancing resilience to food insecurity has changed the way resilience is understood in rural communities of Zimbabwe. Cretney (2016) and Tarhan, Aydin and Tecim (2016) view resilience as the ability of a system to respond, cope and adapt to changes using its *own resources*. Yet in this study community resilience emerged as *sharing resources* with the purpose of enhancing capacity and cushioning each other against disasters. For example, the *Zunde raMambo* produce is shared among vulnerable groups and disaster victims. The smallholder farmers also share farming implements, draught power and human labour during a form of collective work, locally known as *nhimbe.* This strategy is organised at village level where one household invites others to provide labour and draught power for use during ploughing, planting, harvesting, threshing and other construction work. The concept of *nhimbe* is unique in enhancing the coping capacities of communities to improve food security among the poor households. Invitees are neither paid cash nor in kind for performing the work. Instead, the host simply prepares food, refreshments and beer that are consumed by the invitees working on the task called for. This ensures that farming processes or any work are done on time. This differs from paid work performed by multi-lateral agencies and NGOs during emergencies (Hewitt 2013). While the *nhimbe* scheme strengthens solidarity and social cohesion of the community, it also enhances food security among the households without farming implements. The *nhimbe* scheme also acts as a platform for networking when members share expertise, skills and experiences in dealing with disasters.

Draught power is shared in two different ways. Firstly, there is a share-rearing arrangement involving rearing cattle belonging to one household, while using the cattle for various purposes including draught power. No monetary payment is made in this arrangement. Secondly, some smallholder farmers access draught power on an exchange basis. This involves using cattle belonging to households with abundant livestock, for a particular task– ploughing in exchange for labour. The two arrangements are enabling the poor households to cope with food insecurity induced by disasters.

Apart from sharing resources, disaster resilience also involves absorbing environmental stresses and shocks including drought and flood. Absorption embraces the ability to minimise the negative impacts of stresses through appropriate strategies that avoid the negative trajectories associated with the risk (Proag 2014). More important to the absorptive capacity of rural communities is the *Zunde raMambo* scheme. This scheme creates food reserves for disaster victims and other vulnerable people. The traditional leadership has set aside *Zunde raMambo* as a local safety net to cushion the most vulnerable. This initiative has assisted rural communities in drought and flood recovery. The *Zunde raMambo* in Zimbabwe is different from other safety nets in two ways. Rwanda runs safety nets in the form of public works programmes where participants are paid wages (Alderman & Yemtsov 2014). Yet the participants of the *Zunde raMambo* scheme are not paid. Rather, the participants willingly offer their labour and inputs to help those in need. Alderman and Yemtsov (2014) further posit that safety net programmes in China were initiated at national level and provided an element of a broader development policy effort. Although there is no empirical evidence of elements of development policy related to the *Zunde raMambo* scheme in Zimbabwe, there is evidence that the scheme is a local initiative that is enhancing resilience to food insecurity.

In Sri Lanka, Minamoto (2010) researched on the relationship between the people’s perception of livelihood recovery following a tsunami disaster, and social capital to seek more effective disaster support. Results of that study revealed a strong aspect of ‘elite capture’, which was a dark side of collective action with semi-forced participation. This differs from what emerged in this study about social capital. The social capital during the *Zunde raMambo* and *nhimbe* schemes is voluntary and non-coercive. It has both bonding and bridging effects among the community members. While the bonding effect refers to strong connections and relationships between families and friends, largely based on shared identity, the bridging effect describes the relationships across groupings (Macdougall, Gibbs & Clark 2014). The bonding effect in this study is seen in the distribution of the *Zunde raMambo* proceeds among vulnerable members. The bridging effect is witnessed when community members assist each other regardless of tribal background and political and religious affiliations, among others. Both the *Zunde raMambo* and *nhimbe* schemes improve the absorptive capacity of the community to save human lives from food insecurity induced by drought and flood. The *Zunde raMambo* scheme creates food reserves for vulnerable people. The *nhimbe* scheme ensures that farming processes are done on time. While the *nhimbe* scheme strengthens community social cohesion, it also enhances food security among households without farming implements. The *nhimbe* scheme also acts as a platform for networking and sharing expertise, skills and experiences in dealing with food insecurity. Such networks have the potential to generate far more positive outcomes and inclusive benefits across and between villages exposed to flood and drought disasters. They allow people to connect with others outside of their established socio-cultural networks.

## Conclusion

Central to this article is the recognition that traditional institutions are building resilience to food insecurity induced by climate-related disasters. To fulfil this task, traditional institutions have initiated the *Zunde raMambo* and *nhimbe* schemes as an informal safety net, as well as the arrangements of accessing and sharing draught power. The three schemes are evidence of community reorganisation or change in response to food insecurity, with active participation of the communities at risk. They are a form of absorptive capacities enabling the community to cope with food insecurity. This has resulted in bonding and bridging effects within and across communities. Expanding the schemes to other rural areas may enhance the coping and absorptive capacities of rural communities to food insecurity.

## References

[CIT0001] Ainuddin S. & Routray J.K, 2012, ‘Community resilience framework for an earthquake prone area in Baluchistan’, *International Journal of Disaster Risk Reduction* 2, 25–36. https://doi.org/10.1016/j.ijdrr.2012.07.003

[CIT0002] Alderman H. & Yemtsov R, 2014, ‘How can safety nets contribute to economic growth?’, *World Bank Economic Review Advance*, 28, 1–20.

[CIT0003] Ali F.M.M. & Jones K, 2013, ‘Negotiating community resilience in the city in a time of political change and deficit reduction’, *International Journal of Disaster Resilience in the Built Environment* 4(1), 9–22. https://doi.org/10.1108/17595901311298973

[CIT0004] Banerjee R., Kamanda J., Bantilan C. & Naveen P.S, 2012, ‘Exploring the relationship between local institutions in SAT India and adaptation to climate variability’, *Natural Hazards* 65, 1443–1464. https://doi.org/10.1007/s11069-012-0417-9

[CIT0005] Berman R., Quinn C. & Paavola J, 2012, ‘The role of institutions in the transformation of coping capacity to sustainable adaptive capacity’, *Environmental Development* 2, 86–100. https://doi.org/10.1016/j.envdev.2012.03.017

[CIT0006] Birkmann J, 2006, *Measuring vulnerability to promote disaster-resilient societies, conceptual frameworks and definitions*, Routledge, London.

[CIT0007] Brown H., Peach C., Nkem J.N., Sonwa D.J. & Bele Y, 2010, ‘Institutional adaptive capacity and climate change response in the Congo Basin forests of Cameroon’, *Mitigation and Adaptation Strategies for Global Change* 15, 263–282. https://doi.org/10.1007/s11027-010-9216-3

[CIT0008] Brown H.P.C. & Sonwa D.J, 2015, ‘Rural local institutions and climate change adaptation in forest communities in Cameroon’, *Ecology and Society* 20(2), Article 6.

[CIT0009] Coates T, 2015, ‘Understanding local community construction through flooding: The conscious community and the possibilities for locally based communal action’, *Geography and Environment* 2, 55–68. https://doi.org/10.1002/geo2.6

[CIT0010] Coetzee C., Van Niekerk D. & Raju E, 2016, ‘Disaster resilience and complex adaptive systems theory: Finding common grounds for risk reduction’, *Disaster Prevention and Management* 25(2), 196–211. https://doi.org/10.1108/DPM-07-2015-0153

[CIT0011] Cohen L., Manion L. & Morrison K, 2011, *Research methods in education*, 7th edn, Routledge, London.

[CIT0012] Cretney R.M, 2016, ‘Local responses to disaster: The value of community led post disaster response action in a resilience framework’, *Disaster Prevention and Management* 25(1), 27–40. https://doi.org/10.1108/DPM-02-2015-0043

[CIT0013] Cutter S.L., Barnes L., Berry M., Burton C., Evans E., Tate E. et al., 2008, ‘A place-based model for understanding community resilience to natural disasters’, *Global Environmental Change* 18(4), 598–606. https://doi.org/10.1016/j.gloenvcha.2008.07.013

[CIT0014] Cutter S.L., Burton C.G. & Emrich C.T, 2010, ‘Disaster resilience indicators for benchmarking baseline conditions’, *Journal of Homeland Security and Emergency Management* 7(1), 1–22. https://doi.org/10.2202/1547-7355.1732

[CIT0015] Denscombe M, 2010, *Good research guide: For small scale social research projects*, 4th edn, Open University Press, Maidenhead.

[CIT0016] Ellis F. & Bahiigwa G, 2003, ‘Livelihoods and rural poverty reduction in Uganda’, *World Development* 31(6), 997–1013.

[CIT0017] Fekete A., Hufschmidt G. & Kruse S, 2014, ‘Benefits and challenges of resilience and vulnerability for disaster risk management’, *International Journal of Disaster Risk Science* 5, 3–20. https://doi.org/10.1007/s13753-014-0008-3

[CIT0018] Fois F. & Forino G, 2014, ‘The self-built ecovillage in L’ Aquila, Italy: Community resilience as a grassroots response to environmental shock’, *Disaster* 38(4), 719–739. https://doi.org/10.1111/disa.12080 10.1111/disa.12080 25196333

[CIT0019] Gopalakrishnan C. & Okada N, 2007, ‘Designing new institutions for implementing integrated disaster risk management: Key elements and future directions’, *Disasters* 31(4), 353–372. https://doi.org/10.1111/j.1467-7717.2007.01013.x 18028158 10.1111/j.1467-7717.2007.01013.x

[CIT0020] Government of Zimbabwe, 2001, *Traditional leaders act chapter 29:17*, Pub. L. No. Chapter 29:17, Government of Zimbabwe, Harare, Zimbabwe.

[CIT0021] Hewitt K, 2013, ‘Disasters in “development” contexts: Contradictions and options for a preventive approach’, *Jàmbá: Journal of Disaster Risk Studies* 5(2), 1–8. https://doi.org/10.4102/jamba.v5i2.91

[CIT0022] Jordan E., Javernick-Will A. & Amadei B, 2011, ‘Pathways to communicate recovery and resiliency’, in Engineering Project Organizations Conference (EPOC), Bucknell University, Colorado, USA, August 9–11, 2011, pp. 1–14.

[CIT0023] Kayaga S., Mugabi J. & Kingdom W, 2013, ‘Evaluating the institutional sustainability of an urban water utility: A conceptual framework and research directions’, *Utilities Policy* 27, 15–27. https://doi.org/10.1016/j.jup.2013.08.001

[CIT0024] Kumssa A. & Mbeche I.M, 2004, ‘The role of institutions in the development process of African countries’, *International Journal of Social Economics* 31(9), 840–854. https://doi.org/10.1108/03068290410550638

[CIT0025] Lei Y., Yue Y., Zhou H. & Yin W, 2014, ‘Rethinking the relationships of vulnerability, resilience and adaptation from a disaster risk perspective’, *Natural Hazards* 70, 609–627. https://doi.org/10.1007/s11069-013-0831-7

[CIT0026] MacDougall C., Gibbs L. & Clark R, 2014, ‘Community-based preparedness programmes and the 2009 Australian bushfires: Policy implications derived from applying theory’, *Disasters* 38(2), 249–266. https://doi.org/10.1111/disa.12049 24601916 10.1111/disa.12049

[CIT0027] Mack N., Woodsong C., MacQueen K.M., Guest G. & Namey E, 2005, *Qualitative research methods: A data collector’s field guide*, Family Health International, Research Triangle Park, NC.

[CIT0028] Madamombe E, 2004, *Zimbabwe: Flood management practices – Selected flood prone areas Zambezi Basin*, pp. 1–4, Zimbabwe National Water Authority, Harare, Zimbabwe.

[CIT0029] Majumdar P, 2011, *Research methods in social science*, Viva Books, New Delhi.

[CIT0030] Manyena S.B, 2014, ‘Disaster resilience: A question of “multiple faces” and “multiple spaces”?’, *International Journal of Disaster Risk Reduction* 8, 1–9. https://doi.org/10.1016/j.ijdrr.2013.12.010

[CIT0031] Marashe J, 2014, ‘The African traditional religious landscape: An examination of the role of traditional leaders in the fight against HIV and AIDS in Chipinge, Zimbabwe’, *Verbum et Ecclesia* 35(1), 1–8. https://doi.org/10.4102/ve.v35i1.871

[CIT0032] Mavhura E, 2016, ‘Disaster legislation: A critical review of the civil protection act of Zimbabwe’, *Natural Hazards* 80(1), 605–621. https://doi.org/10.1007/s11069-015-1986-1

[CIT0033] Mavhura E., Manatsa D. & Mushore T, 2015, ‘Adaptation to drought in arid and semi-arid environments: Case of the Zambezi Valley, Zimbabwe’, *Jamba: Journal of Disaster Risk Studies* 7(1), 1–7. https://doi.org/10.4102/jamba.v7i1.144 10.4102/jamba.v7i1.144 PMC6043727 30018754

[CIT0034] Mavhura E., Manyena S.B., Collins A.E. & Manatsa D, 2013, ‘Indigenous knowledge, coping strategies and resilience to floods in Muzarabani, Zimbabwe’, *International Journal of Disaster Risk Reduction* 5, 38–48. https://doi.org/10.1016/j.ijdrr.2013.07.001

[CIT0035] MEA, 2005, *Ecosystem and human well-being synthesis*, Island Press, Washington, DC.

[CIT0036] Minamoto Y, 2010, ‘Social capital and livelihood recovery: Post-Tsunami Sri Lanka as a case’, *Disaster Prevention and Management* 19(5), 548–564. https://doi.org/10.1108/09653561011091887

[CIT0037] Mudavanhu C., Manyena S.B., Collins A.E., Mavhura E. & Manatsa D, 2015, ‘Taking children’s voices in disaster risk reduction a step forward’, *International Journal of Disaster Risk Science* 6, 267–281. https://doi.org/10.1007/s13753-015-0060-7

[CIT0038] Nyamwanza A.M, 2012, ‘Livelihood resilience and adaptive capacity: A critical conceptual review’, *Jàmbá: Journal of Disaster Risk Studies* 4(1), 1–6. https://doi.org/10.4102/jamba.v4i1.55

[CIT0039] Patton M.Q, 2002, *Qualitative research and evaluation methods*, 3rd edn, Sage, London.

[CIT0040] Proag V, 2014, ‘Assessing and measuring resilience’, *Procedia Economics and Finance* 18, 222–229. https://doi.org/10.1016/S2212-5671(14)00934-4

[CIT0041] Rumbach A. & Foley D, 2014, ‘Indigenous institutions and their role in disaster risk reduction and resilience: Evidence from the 2009 Tsunami in American Samoa’, *Ecology and Society* 19(1), Article 19. https://doi.org/10.5751/ES-06189-190119

[CIT0042] Skerratt S, 2013, ‘Enhancing the analysis of rural community resilience: Evidence from community land ownership’, *Journal of Rural Studies* 31, 36–46. https://doi.org/10.1016/j.jrurstud.2013.02.003

[CIT0043] Stringer L.C., Dyer J.C., Reed M.S., Dougill A.J., Twyman C. & Mkwambisi D, 2009, ‘Adaptations to climate change, drought and desertification: Local insights to enhance policy in Southern Africa’, *Environmental Science and Policy* 12(7), 748–765. https://doi.org/10.1016/j.envsci.2009.04.002

[CIT0044] Tandlich R., Chirenda T.G. & Srinivas C.S.S, 2013, ‘Preliminary assessment of the gender aspects of disaster vulnerability and loss of human life in South Africa’, *Jàmbá: Journal of Disaster Risk Studies* 5(2), 1–11.

[CIT0045] Tarhan C., Aydin C. & Tecim V, 2016, ‘How can disaster resilience be built with sustainable development?’, *Procedia – Social and Behavioral Sciences* 216, 452–459. https://doi.org/10.1016/j.sbspro.2015.12.059

[CIT0046] Wilson G.A, 2013, ‘Community resilience, policy corridors and the policy challenge’, *Land Use Policy* 31, 298–310. https://doi.org/10.1016/j.landusepol.2012.07.011

[CIT0047] World Bank, 2000, *World Bank development report 1999/2000: Entering the 21st century*, World Bank, New York.

